# Validating Subjective Ratings with Wearable Data for a Nuanced Understanding of Load-Recovery Status in Elite Endurance Athletes

**DOI:** 10.1186/s40798-025-00958-y

**Published:** 2025-12-10

**Authors:** Lina Spetz, Johan Rogestedt, Rickard Nilsson, C. Mikael Mattsson, Filip J. Larsen

**Affiliations:** 1 Department of Physiology, Nutrition and Biomechanics, The Swedish School of Sport and Health Sciences, GIH, Stockholm, Sweden; 2 Silicon Valley Exercise Analytics (svexa), Stockholm, Sweden; 3 Silicon Valley Exercise Analytics (svexa), Menlo Park, CA USA

## Abstract

**Background:**

The emergence of wearable technology offers enhanced real-time health management, including sleep, recovery, and exercise optimization. Despite their potential to monitor load-recovery parameters in elite athletes, the selection, combination, and interpretation or reliance of metrics in relation to perceived impact remain unclear.

**Objective:**

This study assessed data from three wearables measuring sleep, continuous glucose, and exercise, together with the Profile of Mood State (POMS) dimensions alongside subjective ratings via the Readiness Advisor application (RA app) (Silicon Valley Exercise Analytics, svexa, Menlo Park, California, USA) to evaluate their association and value in load-recovery monitoring.

**Methods:**

Twenty national team endurance athletes, competing at the highest international level, were monitored during one year of training, recovery, and competitions. Data collections were made with Global Positioning System (GPS) watches and heart rate monitors, Ōura rings (Ōura Health OY, Oulu, Finland), continuous glucose monitors, POMS questionnaires and subjective ratings in the RA app.

**Results:**

Significant correlations were found between each RA question and its counterpart in a linear mixed model (r values = 0.39–0.81). However, time series analysis through autoregressive integrated moving average (ARIMA analysis) revealed individual variability.

**Conclusions:**

These findings indicate an influence of external aspects and advocate for a multifaceted approach to the assessment of load-recovery balance, well-being and performance. Moreover, personalized analyses proved more accurate than group averages, emphasizing the need for individualized monitoring. Integrating subjective and objective data appears essential for nuanced understanding of the athlete status, advancing high-performance monitoring and athletic health management.

## Key Points

- This study examines the correlation between nine subjectively rated and objectively measured data parameters collected from 20 national team endurance athletes during one year of regular training and competitions.
- Group level analyses only showed moderate significant correlations between the objective and subjective counterparts in three cases. When accounting for individual differences, all subjective parameters presented moderate to strong associations with their objective counterpart (r values = 0.39–0.81). Partial regression analysis suggests that external factors contribute to the observed variability and limit their interchangeability. This advocates for monitoring regimens to include a combination of markers of both objective and subjective origin, to provide a reliable and nuanced understanding of the athlete situation for informed decision-making.
- Individual-level analyses showed large variability, with athletes exhibiting non-, positive, and negative correlations. These results indicate the requirement of individually adapting both monitoring regimes, assessments and load-recovery management.

## Introduction

 In recent years, commercially available wearables have gained increasing popularity in both sports and everyday life [1–3], enabling users to track a broad range of objective parameters related to training, sleep, and health management [4–10]. These devices may serve as valuable resources for personal health monitoring [11, 12]. Wearables also facilitate real-time remote monitoring of physiological responses to the training stimuli, extending beyond traditional session metrics such as GPS and heart rate (HR) tracking. The concept and techniques of creating digital twins expand within both medical fields and sports [13–17]. Combining wearable data streams and daily subjective parameters with the concept of digital twins may improve athletic development, training periodization, and load-recovery management [14, 16, 18, 19]. To achieve this, it is important to identify and evaluate appropriate parameters for the creation of digital twins.

A human digital twin is a digital representation of an individual to accurately model physiological and psychological response patterns. This is achieved by (a) integrating a multimodal set of data to create a comprehensive individual profile, and (b) enable simulations to assess the effect of various stressors and scenarios [13, 14, 20]. Objective measurements and wearable data, such as HR, sleep duration, and glucose levels, provide critical quantitative data about the body’s physical condition. However, these metrics alone cannot fully capture the complexity of human health. Synthesizing both objective and subjective data allows for more comprehensive insights that, in digital twin models, can reflect the intricate interplay between physiological and mental states and events [21–24]. Developing models of a broader composition of parameters provides a richer, more nuanced understanding of an individual’s overall health, where potential influencing aspects are considered, which could be vital for precision health development [21]. However, interpreting this vast amount of data requires multidisciplinary domain knowledge and careful analyses [9, 13]. Processing continuous, extensive data streams demands accessibility, time, and knowledge for the athlete and coach. Without careful planning and interpretation, there is a risk of facing data overload or excessive data collection not bringing value [1, 12, 18].

In athletes, collecting exercise-related data is common practice, intending to evaluate past exercise sessions and to plan future periods. With the access of wearables, more parameters can now be tracked, adding valuable insights about the state of athletes [4–6]. During periods of increased training, athletes are especially prone to imbalances between load and recovery [25–28].

To monitor the training load, both internal and external load metrics are preferable [5, 29, 30]. External load refers to the physical work performed, whereas internal load refers to the physiological and psychological responses to that work [29]. Although the external load may be clearly outlined in the training plan in terms of session content, various exercise-related or external stressful events may influence the resulting internal load and adaptation [24, 30, 31]. Using objective, biomechanical or wearable-derived data alone may also pose a risk of over-reliance on objective data streams, undermining subjective experience or misinterpretation if data parameters are being examined separately. This is an aspect that may be commonly disregarded, despite athletes expressing a need for a more holistic approach where daily life commitments are considered [32–34]. Nutritional intake, psychological state, mental stress, sleep behavior, genetics, individual profile and current training status may all influence the load-recovery balance [24, 29, 31, 35–37].

Despite the plethora of tools available for measuring physiological outcomes, distinguishing reliable tools and sensitive parameters is crucial. Self-reported experiences of physical and mental load are emerging as simple, cost-effective, and accessible methods, with indications of superior sensitivity and consistency compared to objective measures [32]. Additionally, self-reported subjective ratings may encourage reflection on perceived load, recovery, and stress, fostering awareness of how positive and negative adaptations may be experienced or/and manifested in the individual athlete [38, 39]. As the availability of tools and data-driven models increases, key challenges related to interpreting the collected data appropriately and effectively increase. In this context, the present study aims to constitute a step towards parameter selection by systematically evaluating a set of subjective self-rated parameters through the Readiness Advisor (RA) app’s questions by analyzing their association with objectively measured data as counterparts. Our approach involved a comprehensive assessment of the RA system’s ability to capture relevant physiological and psychological parameters, comparing its outputs to previously validated monitoring tools. Through this data-driven analysis, we sought to identify how well the app’s subjective assessments align with objective measurements. If subjective aspects align with objective, these may serve as more accessible, cost-efficient, and informative for athletes and coaches in a monitoring regimen than adding additional objective measurements. A secondary aim was to explore potential reasons that could explain discrepancies between subjective and objective measurements. From a long-term practical perspective, we strive to develop a feasible monitoring regime, potentially benefiting daily conscious decisions to load and recovery efforts to achieve athletic development and performance.

## Methods

### Study Design and Participants

A one-year observational study was conducted to investigate the variation of subjective and objective markers relative to changes in training load among elite endurance athletes. 20 athletes from four different endurance sports (cross-country skiing, long-distance running, orienteering, and triathlon) gave their written consent to participate in this study. The study consisted of free-living load- and recovery monitoring by wearable technology and subjective rating tools, designed to interfere minimally with the athletes training- and competition schedules. Inclusion criteria required the athletes to compete at the highest international level and be part of the Swedish national team in their respective sports. The cohort consisted of 11 men and 9 women, aged 24.4 ± 3.5 years with a female VO_2_max of 63.8 ± 5.6 and a male VO_2_max of 73.5 ± 2.5 ml/kg/min. During their participation, the athletes placed top three a total of 35 times at major international championships or World Cup competitions, indicating their level of performance.

Free-living data collection was conducted to enable regular training regimens, competitions, and traveling. The monitoring included laboratory-based tests and sampling, but these are outside the scope of this study purpose. Athletes individually registered data of perceived load, stress and general well-being at a daily frequency together with measurements of exercise training, sleep, HR parameters and body temperature. They created individual accounts and approved the conditions in each of the wearable applications; the Ōura app (Ōura Health OY, Oulu, Finland), Supersapiens app (Supersapiens Inc., Atlanta, GA, USA), RA app (Silicon Valley Exercise Analytics, svexa, Menlo Park, California, USA) and the app for their individual GPS watch, if not already actively using it. Invites were sent from the researchers to the athletes to allow for visibility in team dashboards and final data exports. In their RA account, integration of the data streams from Ōura and Garmin was approved to allow synchronized data storage. Periodic assessments of blood glucose control and psychological mood states were aligned with periods of various training loads.

Introduction to all monitoring devices was made upon study entry, either in-person or digitally, to on-board each participant to the daily data collection process. During the first week, the athletes were asked to calibrate their daily subjective ratings to reflect their personal perception range.

### The Readiness Advisor (RA) App

**Fig. 1:**
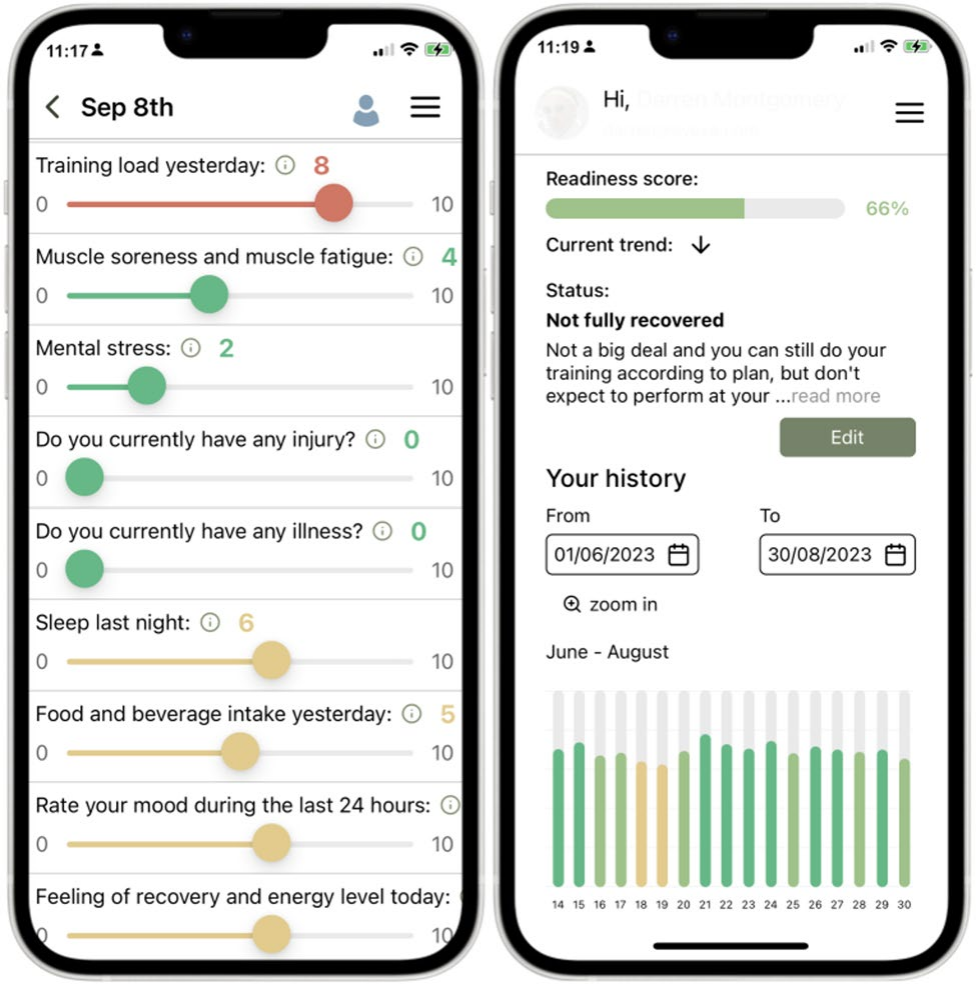
Visual design of the Readiness Advisor (RA) app with the initial page for ratings and the summarized page with the readiness score reinforced by traffic-light coloring

Daily ratings of subjective parameters were made in the RA app (Silicon Valley Exercise Analytics, svexa, Menlo Park, California, USA). It is a free app designed to monitor subjective assessment of load, stress, and recovery in athletes. The app is available for Android and iOS smartphones or as a web version with a coach view functionality. The app is based on nine simple questions created for everyday answer frequency to provide a score of daily readiness, which is a proprietary algorithm created by svexa based on previous research. The result is presented as a percentage readiness score reinforced with a color system ranging from green to red (high recovery status to suboptimal or harmful status), together with suggestions on how to approach the upcoming day’s activities for optimal training and recovery. The readiness score was, however, not included as a data parameter in this study and therefore not mentioned further. This study aimed to validate separate parameters of subjective and objective character, with the RA app providing the questions for subjective ratings that was used in the analyses. Additionally for the study purpose, the athletes were instructed to continue their routine training plan and make alterations as they normally would themselves or together with their coaches, not considering the readiness score as the main basis for load management. The coach view functionality is designed to provide coaches with an overview of their athletes’ current state of recovery, which can be used as an indicator for daily management of upcoming training sessions and maintain desired load-recovery status. During the study period, none other than the researchers had access to the coach dashboards. The questions and result page are presented in Fig. 1.

The participants were instructed to answer the app daily before lunch, receiving a scheduled reminder notification at a selected daily time point in case they had not entered their ratings. The questions within the app are rated between 0 and 10, with each level of the range having an explanation to guide the user when conducting their ratings. Except for the questions related to training sessions, which may imply that both high and low ratings are desirable, four of the statements correlate a high rating as beneficial, whereas the remaining three have a switched direction so that a high numerical rating entails a worse situation. This design forces the athlete to take a moment to think about their response to the question before answering. The app statements are as follows, with an explanation of questions and each step of the rating range presented in Appendix 1.

- Training load yesterday – How high was your training load yesterday?
- Muscle soreness & muscle fatigue – Do you have tired muscles or muscle soreness?
- Mental stress – How high was your mental stress and psychological load yesterday?
- Do you currently have any injury?
- Do you currently have any illness?
- Sleep last night – How well did you sleep last night?
- Food & beverage intake yesterday – How was your food and drink intake yesterday?
- Rate your mood during the last 24 h – Your mood is a combined total rating of your overall feelings.
- Feeling of recovery and energy level today – How recovered do you feel and how is your energy level today?

In the app, female athletes also have a question about timing of menstrual cycle, i.e. [Are you] In menstrual bleeding period? Yes/No, but that question is outside the scope of this study.

### Monitoring Tools and Measurements of Objective Data

#### Exercise Training Measurements

Regular training data registrations were made during the complete period of participation, to capture daily and/or periodic variations. Training and, if possible, competition data were registered with each athlete’s individual GPS watch and connected HR chest strap to provide session frequency, duration, HR, and type of workout. Various brands and models were used, including Garmin (Garmin, Lenexa, Kansas, USA), Polar (Polar, Kempele, Finland), and Suunto (SUUNTO, Vantaa, Finland). The athletes were instructed to wear the watch and HR chest strap during all kinds of training sessions, including strength training, and in as many competitive situations as possible. Each session was instructed to be registered within the correct activity form and to include both warm-up, main part, and cool-down. The athletes were instructed to synchronize their watches to the respective application after each session to store session data on their individual profiles. Except for athletes using Garmin devices, final data exports from their individual profiles were made at the end of the study period and provided to the researchers at that time point. Due to integration and export difficulties, exercise data from two athletes are missing for this study, as well as the first 3.8 months on average for six athletes.

#### Sleep- and Nocturnal Measurements

All athletes were provided with a generation 2 Ōura ring (Ōura Health OY, Oulu, Finland) to be worn every night for measurements of sleep, and nocturnal resting HR (RHR), HR variability (HRV), and temperature deviations (Temp. Dev.). The Ōura ring utilizes infrared photoplethysmography sensors (PPG, sampling rate of 250 Hz) for measuring HR and respiratory rate, negative temperature coefficient sensors (NTC, resolution of 0,07°C) to detect body temperature, and a 3D accelerometer for movement registration (60s resolution), all located in the inside of the ring and adjacent to the palm side of the finger [40]. Based on movement, HR, HRV, and temperature, the combined data is processed by the Ōura sleep staging algorithms to provide estimates of sleep quantity and quality with 96% accuracy for 2-stage classification (sleep, awake) and 79% for 4-stage classification (light, deep, REM, awake) [41]. The athletes were instructed to open the Ōura app each morning to synchronize the data to their Ōura account, thereafter visible to the researchers in a research dashboard. The research dashboard allowed for the full export of all data into CSV files, resulting in separate files for readiness, sleep periods, sleep, activity, and HR-HRV. From these, total sleep time (TST), overnight RHR and HRV, and Temp. Dev. were extracted to be used in this study. HR values were provided in the exports as nocturnal averages, temperature data as a positive or negative deviation from baseline from previous days to weeks, and sleep-related variables as seconds in each sleep stage. Sleep-related variables were recalculated into hours per night.

#### Measurements of Glucose Control

At up to six periods of 14 days each, the athletes were equipped with Continuous Glucose Monitors (CGM) of the type Libre Sense Glucose Sport Biosensor from Abbott (Abbott Diabetes Care, Chicago, USA) connected to the Supersapiens mobile app (Supersapiens Inc., Atlanta, GA, USA). For athletes without an app-compatible phone, the FreeStyle Libre sensor and the FreeStyle Libre reader (Abbott Diabetes Care, Chicago, USA) were used. The athletes were individually instructed on how and when to apply the sensor. Upon instruction, the sensor was mounted with the attached applicator on the back of the shoulder, held in place by an adhesive pad on top of the sensor. When applied, a thin 5 mm filament is inserted right under the skin surface, which measures the glucose concentration in the interstitial fluid with a minute-by-minute frequency for the Biosensor or with a 15-minute frequency for the FreeStyle Libre sensor [42]. With the Biosensor, the data is automatically streamed to the mobile phone application if a Bluetooth connection is maintained and otherwise stored in the sensor with a 15-minute frequency and eight hours of storage time. If connection is lost, a manual scan is required to synchronize the data to the app, and to restore the automatic streaming. As the sensor was applied, it was scanned with the mobile phone to pair them two together, whereafter a one-hour warm-up period was initiated before regular measurements were fully functioning. In the app, glucose readings are presented within the range 54–200 mg/dL (equivalent to 3,0–11,1 mmol/L) with a default normal range set as 70–140 mg/dL (equivalent to 3,9 − 7,8). With the Freestyle Libre and reader, manual scans are required continuously with at least 8 h frequency to not overwrite stored data in the sensor. Application and start-up is identical to the Biosensor. Data is presented every 15 min in the reader, with the units mmol/L and the default normal range of 4–8 mmol/L visualized in the graph.

Two athletes were unable to use the Biosensor and Supersapiens app during their two first measurement periods, whereby the readers were sent to the researchers after the completed periods and manually connected to a computer for export of complete period data to CSV files. In the CSV files, date and time points are presented in one column, glucose values in mmol/L for every 15 min are presented in one, and manual scan values in a third column. Only continuous 15-minute values were used for the analyses. The following CGM periods for these athletes were conducted with the Biosensor and Supersapiens app as the other athletes. Each day was exported separately, with data and time stamp in one column, and glucose values in mg/dL for each 5 min in one column. The Biosensor data was converted to mmol/L from mg/dL by division with 18, to allow for uniform processing and presentation.

#### Mood State Monitoring Via Previously Validated Tools

To provide an assessment of well-being and mood state of the athletes, a digital Swedish version of the psychological rating instrument the Profile of Mood States (POMS) [43] was distributed to the athletes every fifth week via email. The questionnaire consists of 65 statements and is intended to assess mood states in accordance with feelings and experiences during the previous week. Each statement should be rated between 0 and 4, corresponding to how well or not, the individual relates to the statement. The completion of the questionnaire takes approximately 5–10 min. An individual link in the email redirected the athlete to the questionnaire provided in the software Sunet Survey & Report (Artisan Global Media & Artologik, Växjö, Sweden), compatible with both mobile and web versions. The athletes were instructed to answer the questionnaire within 10 days, whereafter the link was closed. All data was exported to Excel files, and thereafter individually processed in POMS: EDITS (MacPOMS: DataMedic AB & Melebo AB, San Diego, USA), where the ratings were calculated to be presented in six dimensions of mood swings; tension, anger, vigor, fatigue, depression, and confusion. From these, two further parameters were calculated: the Total Mood Disturbance (TMD) and the Energy Index (EE-index) [44]. TMD is calculated by subtracting the score for vigor from the sum of the scores for tension, anger, fatigue, depression, and confusion. A higher TMD indicates that mood is negatively affected, and thereby a lower TMD suggests a better mood. EE-index is calculated by subtracting the fatigue score from vigor, which means that a higher score indicates higher vigor [44]. The POMS questionnaire has been repeatedly validated among athletic populations and has been the subject of several reviews and meta-analyses [45–49]. Even though the POMS questionnaire is based on subjective ratings, its extensive set of items and strong scientific validation justified using it as a counterpart to the RA ratings and including it among the objective parameters.

### Data Processing

The processing of the data has been executed partly on svexa’s data processing nodes in the country/region of the athlete and in compliance with all data governing laws, e.g. GDPR, and/or manually in Excel or by using Python with open-source modules to clean, structure, and transfer data to matched and combined Excel files. More information about the svexa nodes and architecture can be found in Appendix 2.

RA data has been cleaned to only include ratings made with experiences fresh in mind. Thereby, a cut-off was set where ratings completed more than 24 h later than the current day were excluded. Sleep rated as 0, which in RA is explained as “Extremely poor, < 1 hour”, was double-checked against Ōura-data and excluded if the TST from Ōura presented several hours of sleep. Four of the questions in RA refer to the experiences from the previous day. These ratings were corrected in placement to align with the matching objective data point. This means that the columns for training load, muscle soreness, mental stress, and food intake were moved one row in the data sheet. Three of the RA questions are of psychological nature - mental stress, mood, and feeling of energy and recovery - and lack a physiologically measurable counterpart. Therefore, the POMS questionnaire, which has been well validated [45–49], was used in the version that requests the statements being considered as the previous week-long experiences of mood states. For the comparative analysis with these three questions, the RA ratings needed to be processed to represent an equivalent period. The average of the past week’s ratings leading up to the day of completion of the POMS questionnaire was thereby calculated and used for the analyses.

Exports of exercise training data were provided in fit. files, with duration, GPS location, and HR as outcome variables. Depending on the watch model and individual settings, data was sampled at different frequencies. The most common in the datasets was a sampling frequency of one second. From the initial export, exercise data was recalculated to present daily exercise as minutes in six different intensity zones. These zones were determined on an individual level from resting-, threshold-, and maximum HR described by Mattsson and Larsen [50]. To gain a simple total calculated training load each day, the duration in hours for each zone was multiplied by 1–6 corresponding to the actual zone and summarized to a final score according to Foster et al. [51]. Several of the athletes preferred not to, or were unable to, use their GPS-watch and HR-chest strap in major competitive situations, resulting in exercise data only from warm-up and cool-down. No measures were taken for this in the initial analyses. However, in the partial regression, we wanted to explore the relationship and ratio between the subjective and objective load. Including days with missing HR data (and probably very high load) posed a risk of misleading results from the analysis, whereby an outlier detection analysis was performed. The ratio between subjective and objective training load was calculated and then processed with the IQR method in python. The 1st and 3rd quartiles were determined, the interquartile range calculated, and thereafter, a lower and upper bound calculated based on Q1-1,5*IQR and Q3 + 1,5*IQR. A load ratio below the lower limit or exceeding the higher limit was excluded from this final analysis, resulting in a total exclusion of 221 days of training from the original 5,875.

Glucose data was stored as separate periods of measurements when all data points had been recalculated to mmol/L. To not include unstable data due to warm-up conditions of the sensors, the first 12 h of each new sensor period was excluded from the data sets. From the 5- or 15-minute continuous values, the percentage time in range (TiR) was calculated on a daily level. With possible gaps in data due to asynchronization or scanning frequency of longer than 8 h, this was calculated as the percentage of measured values within the normal range set at 4–8 mmol/L.

Individual days with missing values in each variable pair were excluded from analysis to not influence the comparisons by imputation of values. For the comparative analyses, we selected the parameters that best reflected subjective and objective equivalents, based on domain knowledge. When a perfect objective counterpart was unavailable, we chose parameters that had potential relevance based on physiological or psychological theory, reflecting a reasonable, data-driven approximation of how subjective experiences might influence measurable outcomes.

- RA Training load compared to calculated training load based on HR data.
  *Reasoning*: This comparison is straightforward, as subjective perceptions of training load are expected to align with measured training load calculated as a function of HR and time [51].
- RA Muscle soreness & muscle fatigue compared to calculated training load based on HR data.
  *Reasoning*: While muscle soreness is a subjective experience, it is often related to the intensity and duration of training, which can be quantified through HR-based training load metrics [21].
- RA Mental stress compared to the POMS tension category and Ōura HRV.
  *Reasoning*: Mental stress is closely tied to physiological responses such as HRV, which measures autonomic nervous system balance [52]. The POMS tension category provides a psychological metric that reflects subjective stress levels [43].
- RA Injury – not included in the analysis due to lack of equivalent parameter.
- RA Illness compared to Ōura RHR and Temp. Dev.
  *Reasoning*: Illness can lead to physiological changes like elevated RHR and body Temp. Dev., making these reasonable objective indicators of self-reported illness [40].
- RA Sleep compared to Ōura TST.
  *Reasoning*: This comparison is direct, as subjective perceptions of sleep naturally are linked to TST. Wearable measured sleep duration is therefore providing an objective counterpart.
- RA Food & beverage intake compared to CGM TiR and Ōura RHR.
  *Reasoning*: Although food intake is subjective, it can affect physiological markers like RHR and glucose regulation, which are captured through CGM with TiR data and RHR measured by the Ōura rings. Inadequate food intake may result in higher glucose variability with more frequent reductions in glucose levels. Meal composition and macronutrient choices can also cause larger glucose peaks, both of which reduce TiR [53]. Because CGM measurements were collected only during specific periods, RHR was included as an additional physiological indicator. Inadequate food and fluid intake can influence metabolism and blood volume, which may lead to both increases and decreases in RHR.
- RA Mood compared to the POMS TMD.
  *Reasoning*: Mood is a subjective experience that can be assessed using the TMD score from the POMS questionnaire, which offers a validated psychological measure of overall emotional state [43, 44, 46].
- RA Feeling of recovery & energy compared to the POMS EE-index.
  *Reasoning*: The feeling of recovery and energy can be compared to the validated POMS EE-index. This index captures an athlete’s balance between vigor and fatigue, providing a measure that aligns with subjective perceptions of recovery and energy [44].

### Data Storage

The RA ratings and the calculated daily readiness are anonymously stored in a PostgreSQL database in compliance with all data governing laws, e.g. GDPR, identified by individual user identification numbers. The data were initially stored via each device’s standard process, locally on the users’ smartphones and subsequently in the cloud infrastructure of the respective manufacturers (Ōura Health, Supersapiens, and Garmin). With participants’ consent, these data were then securely transferred to the research team’s backend platform (“Svexa Lab”) via an API integration or exported directly from a coach dashboard. Participants were informed that their data might temporarily reside on cloud servers located outside the EU, in line with each manufacturer’s terms of service. Ōura-data, as well as Supersapiens data, were also continuously exported from the Coach dashboards to CSV files to ensure access and storage. Additional information about svexa backend with nodes and architecture of data processing can be found in Appendix 2.

### Statistical Analysis Methods

The analyses were made with a stepwise progression of advancements, as a part of the result to explore if individual contribution may provide improved information and usability [54]. In short, we started with traditional correlation analyses of yearly averages, continuing with a linear mixed model for group-level modeling accounting for individual variability, and thereafter included a time series analysis to account for natural variations during the year.

Descriptive statistics and simple Spearman and Pearson correlation analysis were performed using the GraphPad Prism 9 software for windows (version 9.0.2, GraphPad Software, LLC, Boston, USA). All data sheets were analyzed for Gaussian distribution. All analyses were hereafter made using Python with open-source modules.

To account for individual variance as well as accumulated load over time, linear mixed models were used to further investigate the association between subjective and objective variables. Each of the subjective RA variables was set as the outcome variable, with each respective corresponding objective variable as a predictor, considering the random effects of the athletes. This was made separately for each of the eleven variable pairs. The model included both fixed effects, for the predictor, and random effects, for intercepts and slopes, and for each subject. Lastly, a correlation coefficient was calculated between the actual and predicted RA values to assess the predictive power of the models. To develop the linear mixed model further, the ratio between the subjective and objective variables was calculated, which was used as the dependent variable for a partial regression. The remaining RA variables were used as predictors, and the one with the greatest influence on the ratio was identified.

With data collection lasting a full year, the influence of time-related aspects is likely expected, such as training load periodization, and sleep cycle alterations related to seasonal changes in daylight exposure. To explore time series contributions to the comparisons, autoregressive integrated moving average (ARIMA) models were fitted to assess the relationship between two time series, the subjective and objective variable. The correlations were then adjusted for each athlete by determining the best ARIMA order for each comparison, extracting the residuals, and analyzing them using linear and ranked correlations between the residuals, followed by cross-correlation. To visualize the progression of analysis outputs, the adjusted correlations after ARIMA modeling were plotted in a heatmap, with each individual in separate rows and each variable pair in separate columns. The columns were sorted based on the uniformity in the direction of correlations and correlations’ strength to provide a clearer overview of which correlations may be more likely to appeal to athletes on the group level versus which correlations display higher individual variability.

## Results

20 elite endurance athletes at the highest national level completed a year-long participation in the study, resulting in a large number of data points from each measurement tool, as presented in Table 1. The subjective parameters in focus of this study are used in the RA app, with nine daily questions covering perceived training load, muscle soreness and muscle fatigue, mental stress, injuries, illnesses, sleep, food and beverage intake, mood, and the feeling of recovery and energy level. The use of wearables and applications resulted in a total of 5,754 days of subjective 9-item ratings, 6,395 nights with Ōura measurements, 5,875 days with exercise training data, 1,257 days of CGM measurements, and 175 answered POMS questionnaires. Due to synchronization issues, exercise training data from two participants was unable to be used, and CGM data from one participant was lacking.

**Table 1:** Total data volume collected with each monitoring tool during the complete participation period

	Days with RA ratings	Nights with ŌURA data	Days with CGM data	Days with registered exercise training sessions	Answered POMS questionnaires
*Group total*	5,754	6,395	1,257	5,875	175
*Data points per subject (avg. & Std)*	287.7 ± 65.14	319.8 ± 39.97	66.16 ± 18.67	293.75 ± 65.17	8.75 ± 1.34
*Nr. of athletes*	20	20	19	18	20

Data is presented as average values +/- standard deviation

*CGM* Continuous Glucose Monitor, *POMS* Profile of Mood States, *RA* Readiness Advisor

**Fig. 2:**
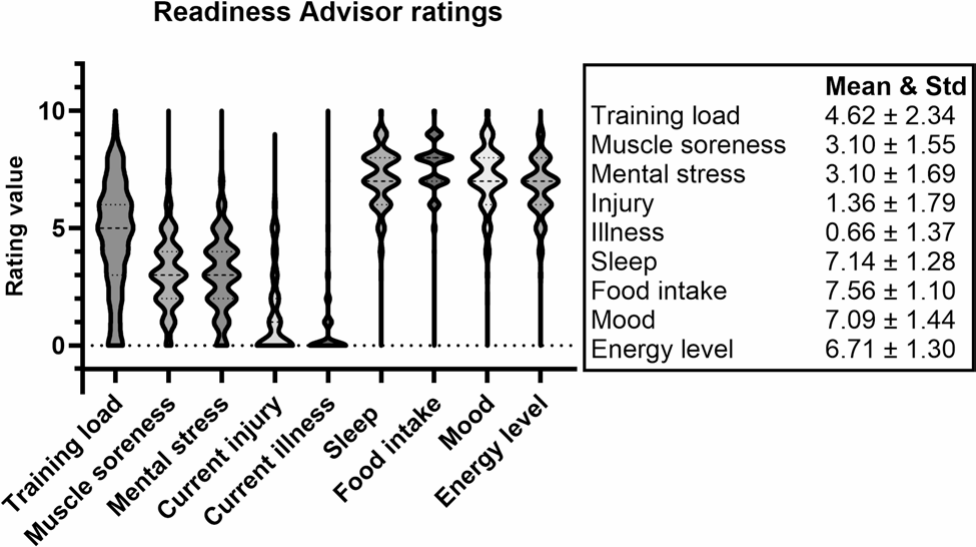
Violin plot of the complete set of Readiness Advisor (RA) data points for all athletes during participation visualized, with descriptive data of mean and standard deviation

For the purpose of comparing subjective and objective variables, one or two corresponding parameters were selected for each of the eight RA questions used for analysis, with the ninth (injury) question excluded due to the lack of an objective equivalent. The analyses were developed step by step, initiated by descriptive statistics to overview the mean and standard deviation of the RA variables and the spread of ratings, presented in Fig. 2. The possibilities, limitations, and differences between statistical methods and analysis were explored with this stepwise process of advancement to gain a better understanding of the associations between objective and subjective variables with an individual aspect.

Std: standard deviation.

### Correlation Analysis between Full-Year Averages

The second step of the analysis was to assess the relevance of absolute values obtained from both objective devices and subjective ratings. In practical sports settings, this initial approach to monitoring parameters can risk rigid interpretations if not contextualized against individual baselines. As an example, there might be an athlete sleeping regularly 7.5 h per night and experiencing this as adequate, whereas another athlete would consider 7.5 h insufficient and thereby subjectively rating these nights far lower. Using TST alone and assuming that the readiness to exercise would be similar for these two athletes would be far from ideal. To investigate this assumption, we calculated the average value for each metric over the entire participation period for each athlete and performed correlation analyses between the subjective and objective counterparts.

Although these yearly averages do not capture the full extent of variations, neither between individuals nor in load metrics relative to training or competitive periods, the analysis revealed several significant correlations, as presented in Table 2. Subjective mental stress was correlated to the tension parameter from the POMS questionnaire as well as subjective mood to the summed score of TMD from the POMS questionnaire. Additionally, the subjectively rated food and beverage intake correlated negatively with RHR measured by the Ōura ring.

**Table 2:** Correlation analysis of full period averages on the group level, with pearson correlation coefficient used for normally distributed data, otherwise spearman’s rank correlation coefficient

RA Question	Objective variable (Data providing device)	Number of athletes	*r*	Pearson *R*^2^	*P* value
*Training load*	Calculated training load (GPS watch + HR monitor)	18	-0.2941#		0.2361
*Muscle soreness* *& fatigue*	Calculated training load (GPS watch + HR monitor)	18	0.01961#		0.9384
*Mental stress*	HRV (ŌURA ring)	20	-0.0075#		0.9749
	Tension (POMS questionnaire)	20	0.5253	0.2459	0.0174*
*Current injury*	-	-	-	-	-
*Current illness*	Temp. Dev. (ŌURA ring)	20	0.2248	0.05054	0.3406
	RHR (ŌURA ring)	20	0.1410	0.01989	0.5531
*Sleep last night*	TST (ŌURA ring)	20	0.1893	0.03585	0.424
*Food & beverage intake*	TiR (CGM)	19	-0.04737#		0.8473
	RHR (ŌURA ring)	20	-0.5946	0.3536	0.0057**
*Mood last 24 h*	TMD (POMS questionnaire)	20	-0.4735	0.2242	0.035*
*Feeling of energy & recovery*	EE-index (POMS questionnaire)	20	0.3347	0.1120	0.1492

# Non-parametric data with Spearman’s rank correlation coefficient. * *p* ≤ 0.05, ** *p* ≤ 0.01

*EE-index* Energy Index, *HR* Heart Rate, *HRV* Heart Rate Variability, *POMS* Profile of Mood States, *RA* Readiness Advisor, *RHR* Resting Heart Rate, *Temp. Dev*. Temperature Deviation, *TiR* Time in Range, *TMD* Total Mood Disturbance, *TST* Total Sleep Time

### Modeling the Group-Level Response

The use of absolute data may be misinterpreted at an individual level if baseline values are not considered. Beyond that, averaged data points only provide a blunt picture without deeper knowledge of the daily variations. An initial overview of absolute data showed variance in both the strength and direction of correlations between individuals. To explore the individual contribution to the analysis, a mixed-effects linear model analysis was developed. We aimed to explore the relationship between the variables in each pair, with the objective parameters as predictor variables and the subjective RA parameter as the outcome variable, while considering the random effects of individual variance.

As presented in Table 3, the correlation between the objective variables and predicted subjective variables was improved in all pairs except for RA food vs. RHR when subject-level variability was accounted for.

**Table 3:** Results from the linear mixed model (LMM) analysis between subjective and objective variables, with adjusted group level r-values accounting for individual variance

RA question	Objective variable	Number of data points	Spearman *r* *of the LMM model* (actual vs. predicted)	P value *for RA question vs. * *objective variable*	Strongest RA predictor on the subjective-objective ratio (Coefficient)
*Training load*	Calculated training load	3,860	0.7018	< 0.0001****	Mental stress (0.15)
*Muscle soreness* * & fatigue*	Calculated training load	3,860	0.5399	< 0.0001****	Mental stress (0.22)
*Mental stress*	HRV	4,882	0.5618	0.096	Mood (0.21)
	Tension	135	0.7853	< 0.0001****	-
*Current injury*	-	-	-	-	-
*Current illness*	Temp. Dev.	4,973	0.4226	< 0.0001****	-
	RHR	4,919	0.3931	< 0.0001****	Training load (0.19)
*Sleep last night*	TST	4,975	0.6974	< 0.0001****	Energy level (0.26)
*Food & beverage intake*	TiR	1,041	0.4693	0.628	Mood (0.14)
	RHR	4,887	0.4323	0.006**	Illness (0.13)
*Mood last 24 h*	TMD	146	0.8165	< 0.0001****	-
*Feeling of energy* * & recovery*	EE-index	146	0.7935	0.009**	-

The right column presents results from the partial regression analysis, with RA variables as predictors for the ratio between the subjective and objective variables in each pair

** *p* ≤ 0.01, **** *p* ≤ 0.0001. Predictors lacking for the ratios for mental and tension, illness and Temp. Dev., mood and TMD, and Energy and EE-index due to insufficient data and/or low variance in data values, therefore not contributing meaningfully to the model

*EE-index* Energy Index, *HR* Heart Rate, *HRV* Heart Rate Variability, *POMS* Profile of Mood States, *RA* Readiness Advisor, *RHR* Resting Heart Rate, *Temp. Dev.* Temperature Deviation, *TiR* Time in Range, *TMD* Total Mood Disturbance, *TST* Total Sleep Time

### The Influence of Time-Related Variance during a Year Explored with ARIMA Analysis at the Individual Level

**Fig. 3:**
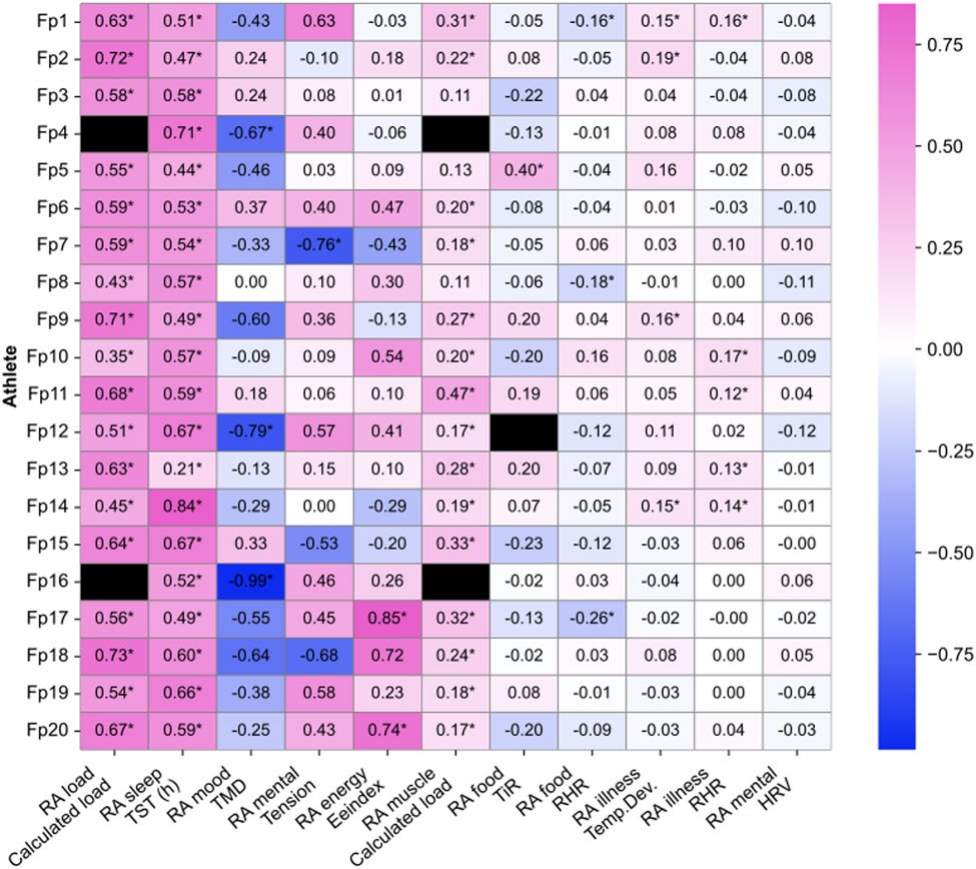
A heatmap presenting the linear mixed model that has been developed to individual level and with Autoregressive Integrated Moving Average (ARIMA) analysis to explore the possible influence of time series variability and time lags in the variables. The stronger correlations towards the left side of the heatmap. R-values are presented in each cell, with an asterisk * for significant correlations. *EE-index* Energy Index, *HR* Heart Rate, *HRV* Heart Rate Variability, *POMS* Profile of Mood States, *RA* Readiness Advisor, *RHR* Resting Heart Rate, *Temp. Dev.* Temperature Deviation, *TiR* Time in Range, *TMD* Total Mood Disturbance, *TST* Total Sleep Time

While group-level models are a valuable starting point for identifying general patterns, the strength and relevance of these relationships can vary significantly between individuals. This variation is crucial to acknowledge when developing digital twin models, which aim to capture the unique physiological and psychological characteristics of each person. To create effective digital twins, it is essential to map out individual patterns and responses, as these often differ from group averages. Therefore, the next step after the linear mixed model was to do individual analysis and explore the potential influence of time series variability and lags of the dependent variable. ARIMA models were fitted for each variable pair, and to highlight the variability and uniqueness in each individuals’ physiological responses the results are illustrated in the heatmap in Fig. 3.

Our findings indicate that the correlation between subjective training load and calculated training load yielded the highest r-values across subjects, though the range was broad, between 0.35 and 0.73. This suggests that the influence of additional factors affecting subjective ratings of training load varies considerably between individuals. Another notable example is the lack of correlation between RHR and illness. RHR has traditionally been used as an indicator of an athlete’s readiness to train, with elevated morning RHR levels above a certain threshold signaling the need for rest. In our study, we found that RHR was significantly correlated with illness only in 6 out of 20 athletes. Also, HRV and subjective mental stress did not present a significant correlation. Despite being a popular objective marker, in this data set, with knowledgeable participants highly attuned to their bodies, the correlation values between HRV values and mental stress are remarkably low for all athletes.

## Discussion

This study offers one of the first longitudinal assessments of international-level elite endurance athletes that integrate both subjective self-ratings and objective data from wearables over an extended period of time. While prior studies have focused on shorter-term [55], single-athlete observations [22], or isolated metrics such as HRV or sleep quality [56, 57], our research applies daily or periodic monitoring of multiple parameters across a full year of training and competition. This approach provides a comprehensive view and deeper understanding of how physiological and psychological states evolve and vary on both inter- and intra-individual levels.

Our primary objective was to compare a set of subjective ratings collected through the RA app with corresponding objective variables measured by wearables or established questionnaires, as a step towards conscious selection of parameters for monitoring regimes. In recent years, several studies have indicated the importance and value of including subjective parameters in a monitoring regimen [21, 38, 39, 58], even with indications of superior sensitivity and consistency in relation to objective measures [32]. While we observed significant correlations between subjective and objective parameters, these correlations were not perfect, certainly not at the individual level. These results suggest that external factors, outside each separate comparison, such as psychological stress, environmental conditions, and/or individual variability may influence the disparities between objective measurements and subjective experiences [38]. Also, it indicates the importance of not making load-recovery decisions based on separate parameter evaluations distinguished from a broader perspective.

Elite athletes frequently challenge the balance between load and recovery, where exceeding the load without accommodating adequate recovery can jeopardize both short-term performance and long-term health [59–61]. Integrating assessments of subjective and objective markers could not only increase the accuracy of load-recovery monitoring but also enhance athletes’ ability to reflect on and manage their perceived situation [38, 39]. Both too limited and too extensive selection of monitoring parameters poses a challenge. With a minimal selection, crucial aspects could remain undetected, or data being misinterpreted, whereas large-scale sets of parameters require extensive processing and interpretation before being useful [1]. Although data collection from multiple sources is common in elite sports, the challenge lies in effectively processing and interpreting this data [12]. Generic, group-level recommendations, such as standardized sleep or recovery times, often fail to reflect the highly individual and dynamic nature of these responses [30, 62]. Our findings emphasize the significant role external factors play in the delicate balance between training load and recovery, making it crucial to consider what individual athletes experience outside the arena of physiological exercise training.

Conducting regression analysis on longitudinal data from multiple subjects is complex, as this data is inherently non-independent and tends to exhibit autocorrelation and/or multicollinearity. Moreover, elite athletes typically organize their training into micro-, meso-, and macrocycles, where the training load varies with daily, weekly, or monthly frequencies [63]. Other parameters such as mental stress, mood, or sleep may vary with different frequencies, which in turn may interact with each other with both positive and negative outcomes [23, 24, 31, 34]. In unfortunate situations, increased load and stress in various aspects may occur simultaneously, magnifying the total load [23, 24, 31, 35]. These variations introduce time-dependent changes in the data, which must be carefully accounted for in the analysis to ensure accurate interpretations and results, again considering multiple aspects.

We initiated our analysis by creating a single data point per athlete for each objective/subjective pair, the average in each variable, to assess the correlation between the absolute values. For instance, we questioned whether an athlete with a lower average sleep rating also experienced less TST, or whether athletes who accumulate more training load perceive their subjective training load as more strenuous. As anticipated, most objective/subjective pairs did not exhibit significant correlations. This suggests that individual baselines are more critical than the absolute numbers [1, 54]; for example, one athlete might feel well-rested after only 7 h of sleep, while another may require 9 h. Unexpectedly, a few parameter pairs did show significant correlations. Notably, there was a negative correlation between RHR and subjective food and fluid intake; individuals with lower ratings on food intake generally had higher RHR. The physiological underpinnings of this observation warrant further investigation in relation to a more specific examination of what the rating is based upon, i.e. if the increased RHR is a function of inadequate fluid intake and therefore a reduction in blood plasma [64] or other mechanisms.

As the next step, we employed a linear mixed model approach to account for individual variations in both baseline values and slopes of the correlations. This allowed us to explore deeper and gain insights from the rich dataset. As shown in Table 3, the relationship between actual and predicted subjective variables exhibited relatively high r-values, indicating that the model’s predictions moderately aligned with the subjective ratings. The p-values were statistically significant across most variable pairs, with the notable exceptions of RA food and TiR for blood glucose, as well as mental stress and HRV. In recent years, HRV has gained increased attention in media and marketing campaigns as a parameter related to stress levels. Several producers of wearables explain HRV as a potential assessment of the autonomic nervous system, with its two branches of sympathetic and parasympathetic activity in turn explained together with “fight or flight” or “rest and digest” dominance [52]. The findings in this study do not align with this, as the subjective mental stress was not significantly correlated with HRV and also with the r-values varying between positive and negative direction.

The relationship between subjective food rating and TiR for blood glucose proved to be complex. Four subjects exhibited a positive correlation, six showed a negative correlation, and for the remaining subjects, TiR did not appear to be a significant predictor of food ratings. Food-related questionnaires are challenging to interpret, as 24-hour recall of food intake is often only weakly correlated with actual intake [65]. Additionally, athletes’ perceptions of what constitutes “good” or “bad”, nutritious or energy-rich food intake may differ. Endurance athletes, who are sometimes prone to restrictive eating behaviors due to body weight being related to energy consumption and performance outcomes, may report higher food ratings when they adhere to a restricted diet, which could influence the correlation between subjective intake and actual meal compositions. Although diet quality and carbohydrate intake may be associated with fluctuations in glucose control [53], this could not be determined in our analyses.

The training load was calculated as a function of HR and duration [51]. It’s important to note that HR primarily reflects aerobic exercise [64]. Therefore, shorter, sprint-like activities, which rely more on anaerobic energy systems, may not be accurately reflected by HR. This also applies to strength-based sessions. These activities can be perceived as highly stressful and contribute to accumulated subjective load, even though the HR method may not fully capture their intensity. This outcome underscores the nuanced interplay between subjective perceptions and objective measurements, where objective data alone could underestimate perceived load.

To improve the precision of longitudinal monitoring and subsequent load-recovery management, it is crucial to select relevant data inputs and consider individual variability [9, 12, 54, 66]. While the group-level linear mixed model demonstrated satisfactory accuracy, the true potential of longitudinal analysis lies in creating personalized models, ‘digital twins’, that can be continuously updated with incoming data, capturing the dynamic, time-dependent structures inherent in the dataset [13, 14, 66]. To achieve this, we analyzed each subject individually, fitting ARIMA models to remove time-series trends and subsequently calculated correlations on the residuals. This approach allowed for a more refined understanding of underlying patterns by accounting for temporal dependencies on a per-subject basis.

With the current advancements in wearable technology and machine learning, there is a broad range of accessible wearables and tools that may provide an increased possibility of tracking parameters related to exercise training, sleep, glucose tolerance, etc. [1, 5–7, 9, 14, 16]. Although wearables create and provide new possibilities for monitoring, potential disadvantages or risks with extensive streams of personal information need to be considered. The anonymity and privacy of data, restrictions, or availability require clear review and careful approval initially to ascertain that the data streams are not used externally in a way that intrudes on privacy [67]. But regardless of the possibilities of receiving live streams of data, the knowledge and possibilities of interpreting these parameters to gain a beneficial evaluation is the key factor [1, 9], which, to the best of our knowledge, is still lacking. An extensive monitoring regime with several tools that require daily engagement may be considered both unnecessary, excessively time-consuming, and/or exhausting. Especially if proper use and valuable outcomes are not obtained. Additionally, multiple data streams of objective parameters may diminish the reliance on perceived and subjective parameters and imply an over-reliance on technological devices and models [1, 66]. This, together with the indications of subjective assessments as superior [32], the rapid development of wearables may seem contradictory and attract to excessive trust in objective data and diminish perceived load and recovery status [66]. A balanced combination of objectively measurable and subjectively rated parameters may, therefore, constitute a golden mean, incorporating the beneficial aspects of both raw data and perceived load-recovery aspects that only the individual her-/himself can experience.

Although the correlations between the rated values in the RA app and their corresponding objective measures were not perfect, the majority of correlations identified were statistically significant. Our interpretation of these findings is that no single parameter can offer a complete and reliable assessment of an athlete’s load-recovery balance. Correlations of non-significance or significant but weak correlations may imply that the parameter individually cannot reflect the picture fully, but in combination with others still provide valuable information to be considered in practice, or something that the individual athlete could reflect about. Regardless of parameter, strength of correlation, or significance level in statistical analyses, potential load-recovery management should be made with care, considering the data in combination with the purpose and objective of the current period. Further studies are therefore required to investigate how to conduct load-recovery management based on data streams as the ones being explored in this study, and how individual thresholds may affect adaptation.

The results from this study highlight the complexity of human physiology and, by extension, athlete health and performance monitoring. Subjective perceptions of various load-, recovery-, and well-being aspects cannot be fully captured by objective metrics alone, underscoring the requirement of a multifactorial approach in athlete monitoring. Relying solely on subjective assessments would instead limit the ability to evaluate, compare and plan training periods for future development and optimization. Developing a monitoring regimen that includes sensitive and reliable parameters, considering multiple dimensions of load and recovery to form a complete picture of an athlete’s state could be highly beneficial for maintaining athletic performance and overall well-being. The RA app, with its combination of the nine subjective rating statements, is designed with this multidimensional perspective in mind. It may provide athletes with a practical, easily accessible monitoring tool that not only prompts reflection on perceived emotional, physiological, and mental responses but also offers guidance to consider for managing exercise training and recovery. It could be a valuable asset in combination with existing objective measurements to nuance the assessment of recovery state, or as an alternative to objective measurements in budget constrained situations where extensive measurements are impossible. Based on the results showing individual variance, we do believe in and propose athletes and coaches to select monitoring parameters on an individual level to obtain valuable and actionable insights for the specific athlete and sport domain.

Current technological aids require several devices to be used to receive data streams of various parameters. If careful selection of device sensors is made, beneficial development of wearables would include additional combinations of sensors to minimize the number of different devices to wear, synchronize, and interpret on a daily basis. Although the device size may pose a problem with a more large-scale combination of sensors, sport-specific combinations could expand the market of wearables that match certain populations. One example could be within long-distance running, where GPS, HR, and lactate measurements are commonly used, and CGM is increasing. Currently, this requires three separate devices that include wear and charging, and each with an application that demands synchronization and evaluation. If combined, time and effort could be minimized as well as the availability of merged data overviews. Moving forward, studies investigating various models of data merging, processing, and analyses as well as succeeding interpretation and guidance, should be explored practically to obtain knowledge about how different athletes respond to various thresholds of load-recovery recommendations.

## Conclusion

To facilitate reliable load-recovery management in athletes, a multidimensional monitoring regime is beneficial. The results presented here, with moderate to strong correlations between objective and subjective parameters that are influenced by external aspects, highlight the importance of including subjective and objective aspects in monitoring methods, such as combining an objective measure of training load with a subjective rating of training load to acknowledge external influence. Subjective experiences in combination with objective and wearable measured parameters provide a more nuanced and comprehensive overview of the athlete situation. Markers related to exercise training, sleep, stress, mood, nutrition and recovery, do all affect the state of the athlete and provide a basis for decision-making processes. This study also presents individual variability in correlation strength and direction. Therefore, the findings of this study point towards the conclusion that a personalized monitoring regime and profile should be developed for each athlete, including measurements and parameters selected and based on individual response patterns and the daily situation.

## Figures and Tables

**Fig. 4:**
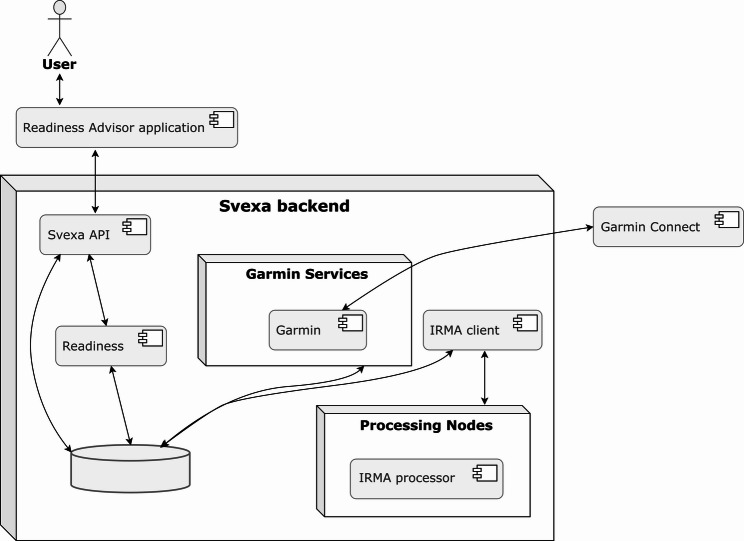
High level architecture of svexa’s data processing system

## Data Availability

The data collected for this study originates from a group of high-performance, international level elite athletes in individual endurance sports. Access to raw data may pose a risk of identification of individual athletes. Additionally, the extensive set of data during a full year of training and competing may uncover competitive advantages, strengths, and weaknesses. Therefore, data will not be made publicly available to ensure the anonymity of the study participants. Concerning transparency, corresponding authors may provide a subset of aggravated group-level data to verify the dataset and analyses to qualified researchers upon reasonable request to ensure participant confidentiality. The data collection for this study included several wearable devices, some of which the athletes were already using and some that were introduced as an addition to fulfill the study’s purposes. When initiating study participation, accounts for each wearable were created individually by the athletes upon agreement for data storage was approved, likewise, integration of data streams from Garmin and Ōura to their RA account was approved.

## Appendix 1

The Readiness Advisor app

*Training load yesterday:*

How high was your training load yesterday? Your total training load is a combination of duration and intensity, and combined score if you do several sessions during one day.

0 No activity at all
1–2 Easy training, physical activity
3–4 Training to maintain level, no improvement expected
5–6 Normal hard (heavy) training, full training session
7–8 Harder than normal, e.g. longer or harder sessions
9–10 Maximal training load, conscious overtraining

*Muscle soreness & muscle fatigue:*

Do you have tired muscles or muscle soreness? Consider your muscles’ feeling today.

0 Muscles are perfectly fine, feels bouncy and explosive
1–2 No fatigue/pain. Normal muscles
3–4 Slight muscle fatigue/pain
5–6 Moderate muscle fatigue/pain
7–8 Pronounced muscle fatigue/pain
9–10 Extreme muscle fatigue/pain

*Mental stress:*

How high was your mental stress and psychological load yesterday? Mental, or cognitive, stress is a combined total rating of everything that affects you, incl. sports performance, work or academic stress, social life stressors, and/or any changes. Travel stress, learning new skills, and giving presentations or talking to media shall also be taken into account.

0 No stress or effort at all
1–2 Very little conscious mental effort. Almost everything known and automatic
3–4 Some mental effort and stress. Complex but familiar activities and situations
5–6 High mental effort. Moderate complexity due to some uncertainty, unpredictability, or unfamiliarity
7–8 More than normal, extensive stress, mental effort and concentration. New, unknown and/or complex activity requiring full attention
9–10 Maximal stress and effort. Completely exhausting.

*Do you currently have any injury?*

An injury can be an overuse injury, such as muscle strains or acute injuries such as sprained ankles or fractures.

0 Nothing affecting me, completely healthy
1–2 Very minor issue that do not affect training
3–4 A small injury that needs care but does not hinder training
5–6 Injury that require adjustment in training
7–8 Painful injury that affects movement pattern. Normal training impossible
9–10 Painful/serious injury that requires painkillers or affects daily life. No training except early rehab is possible

*Do you currently have any illness?*

An illness can be for example a common cold, sore throat, fever or any other infection affecting your wellbeing.

0 Nothing affecting me, completely healthy
1–2 Very minor issues that do not affect training
3–4 A runny nose, itchy throat or headache that does not hinder training
5–6 A common cold or sore throat that require adjustment in training
7–8 A fever or other infection that stops you from training
9–10 High fever or serious infection/influenza

*Sleep Last Night*

How well did you sleep last night? Sleep is rated as a combination of quantity and quality. The benchmark for the rating is the number of hours slept, but can be shifted up or down if the quality was better or worse than normal.

0 Extremely poor, < 1 h
1–2 Very poor sleep, just a couple of hours
3–4 Poor, anxious, too short sleep
5–6 Insufficient sleep, tired when raising
7–8 Good sleep, 7–8 h normal sleep
9–10 Very good sleep, > 9 h, woke up without alarm

*Food & Beverage Intake Yesterday*

How was your food and drink intake yesterday? Perfect food and drink intake means three main meals + 1–2 snacks yesterday, with good timing, desirable nutrients, and sufficient fluids.

0 No food at all
1–2 Major issues that should disqualify from hard training
3–4 Large flaws, e.g. only 1 proper meal during travel day. Fluid status: Dark yellow urine
5–6 Medium flaws, e.g. missed meals, or mainly junk food
7–8 Minor flaws, e.g. suboptimal timing, nutrients or not enough fluids
9–10 Perfect food and fluid intake. Fluid status: Light yellow urine

*Rate your Mood during the Last 24 h*

Your mood is a combined total rating of your overall feelings. The mood and stress level is a combined total rating that can be affected by everything from sports performance, work or academic stress, social life stressors, and/or any changes.

0 Angry, worn out, depressed or lethargic
1–2 Not good at all, irritated, anxiety and/or depression symptoms
3–4 Not good, irritated, angry, doesn’t feel balanced
5–6 Somewhat stressed, easily irritated and annoyed
7–8 Fine, happy, can easily handle stressors, “in balance”
9–10 Very good, very happy, clearheaded, completely calm, relaxed, powerful, stable and in harmony

*Feeling of Recovery & Energy Level Today*

How recovered do you feel and how is your energy level today? Taking both physical and mental aspects into account, rate your own feeling of recovery and energy level.

0 Terrible, no energy, requires several days of rest
1–2 Not at all recovered, need resting day, and energy boost
3–4 Not well recovered, minimal energy, only able to do light training
5–6 Moderately recovered, lacking a bit of energy, hard to find the energy to train
7–8 Well recovered, energy to train as usual
9–10 Perfectly ready to go, fully recovered, full of energy

.

*In Menstrual Bleeding Period*

Yes/No.

## Appendix 2

The svexa nodes and architecture of data processing system

The svexa nodes are deployed in a highly scalable manner that allows simple and repeatable processing of data in a controlled environment. The generation of a new version of the athlete is initiated as soon as there is new data available. Data that enters the system, either by the user directly or from a third party like Garmin, will place the user in a work queue. The IRMA client is responsible for processing this work queue and uses the IRMA processors to execute the calculations. This ensures a highly scalable structure and an architecture where no data, except the uploaded data for the specific execution session, can be accessed by the IRMA processors. Activities from Garmin are automatically pushed to svexa’s Garmin integration service when new data is available. When the data processing is done, the IRMA processor puts all the processed data in a compressed and encrypted file. This file is then saved in the data storage and can only be read by granted services and/or persons. A visual overview of the architecture is presented in Fig. 4.

## Code Availability

The underlying code for this study is not publicly available but will be provided upon request to the corresponding authors.
